# Focusing on The Association Between Cavum Septum Pellucidum and/or Cavum Vergae and Clinical Symptoms and Drug Therapy in Patients with Schizophrenia

**DOI:** 10.31083/AP40036

**Published:** 2025-04-28

**Authors:** Nermin Gündüz, Ahmet Yosmaoğlu, Erkal Erzincan, Gülçin Şenyuva

**Affiliations:** ^1^Department of Psychiatry, Faculty of Medicine, Halic University, 34060 Istanbul, Türkiye; ^2^School of Medicine, Department of Psychiatry, Uskudar University, 34768 Istanbul, Türkiye; ^3^Department of Psychiatry, NP Istanbul Brain Hospital, 34768 Istanbul, Türkiye; ^4^Free Psychiatry Office, 34744 Istanbul, Türkiye; ^5^Department of Psychology, Faculty of Science and Literature, Halic University, 34060 Istanbul, Türkiye

**Keywords:** schizophrenia, cavum septum pellucidum, cavum vergae, psychotic disorder

## Abstract

**Objective:**

Septum pellucidum is a thin midline brain structure located in the anterior brain, running in a median-sagittal or midsagittal direction. This study aims at testing whether cavum septum pellucidum (CSP) and cavum vergae (CV) could predict clozapine pre- scribing in patients with schizophrenia. This study also assesses the relationship between CSP/CV and some clinical findings in patients with schizophrenia.

**Methods:**

190 patients diagnosed with schizophrenia who underwent neuroanatomical evaluation with magnetic resonance imaging during inpatient treatment were included in the study. A personal data form, Positive and Negative Syndrome Scale (PANSS) were given to each patient at admission and discharge. The presence or absence of CSP/CV was recorded as “yes” or “no”.

**Results:**

The presence of CSP/CV was found to be associated with the number of hospital admissions, the number of electroconvulsive therapy sessions received, PANSS total score at admission, PANSS total score at discharge and clozapine use. In the logistic regression model created, the presence of CSP and total PANSS score were found to predict clozapine prescribing (respectively *p* = 0.001, *p* = 0.016). The Nagelkerke’s R^2^ value was found to be 0.167.

**Conclusions:**

This study holds the distinction of being the first in the field to investigate the relationship between clozapine prescribing and the presence of CSP/CV in schizophrenia patients. There is a need for longitudinal-cohort studies that can better express effect to identify the conditions associated with CSP/CV.

## Main Points

1. Detection of the presence of CSP/CV in patients with schizophrenia during follow-up may be important in understanding the clinical course of the disease.

2. Studying the role of CSP in clinical course of schizophrenia, we found that the presence of CSP/CV was found to be associated with the number of hospital admissions, PANSS total score and clozapine use.

3. The presence of CSP and total PANSS score were found to predict clozapine prescribing.

## 1. Introduction

Schizophrenia is a mental disorder characterized by a wide range of symptoms/symptom clusters and variable clinical courses, or rather a group of disorders [1]. Literature in the field emphasizes that schizophrenia can not be classified as a categorical chronic illness [2]. For many years, there has been a debate about whether schizophrenia should be treated as a separate and autonomous nosological entity or be seen as a ‘schizophrenia group’ or ‘schizophrenia spectrum’. Discussions surrounding the consideration of schizophrenia as a separate nosological entity are a result of the varying clinical symptoms of the disease, its heterogeneous course, and, most importantly, its aetiology, which is not fully understood despite various pathogenetic hypotheses [3].

Despite over a century of ongoing research, the aetiology of schizophrenia still remains uncertain. Aetiological theories proposed over the years tend to complement each other rather than definitively explain the formation of the illness. It is widely accepted that the interaction of environmental and biological factors plays a crucial role in the development of this severe cognitive impairment. The neurodevelopmental model suggests that schizophrenia is a behavioral consequence of a deviation in neurodevelopmental processes that begins long before the onset of clinical symptoms, during prenatal, perinatal, and postnatal periods, extending into adolescence and results from a combination of environmental and genetic factors [4, 5, 6]. Recent longitudinal brain imaging studies conducted in both early and adult-onset populations have shown that brain changes are more dynamic than previously thought to be [7]. Some researchers, based on observed changes that occur before, during, and after the onset of the illness, have suggested that there are disruptions or disturbances in early (prenatal and perinatal) and late (postpubertal) neurodevelopment for some individuals who develop schizophrenia [8].

In recent years, data related to cavum septum pellucidum (CSP) have begun to draw attention in schizophrenia and neuroimaging research. Septum pellucidum (SP) is a thin midline brain structure located in the anterior brain, running in a median-sagittal or midsagittal direction. It is a component of the limbic system and extends between the genu and body of the corpus callosum, forming the medial walls of the lateral ventricles. SP serves as a transitional station in the limbic system, connecting the hypothalamic autonomic system to the hippocampus, amygdala, and habenula, while also regulating the brainstem reticular formation [9, 10]. Although a small structure, it can exhibit various congenital or acquired anatomical variants [11, 12]. CSP is present in all fetuses and premature infants and closes within one month after birth in 15% and within six months in 85% of cases [9]. However, the presence of CSP later in life may reflect developmental abnormalities in the surrounding structures, such as the corpus callosum and hippocampus. Therefore, CSP can be considered a marker of limbic system dysgenesis, a form of midline abnormality, or both [13]. Initially widely used to study fetal development [14], CSP may be considered a neurodevelopmental marker in later stages of life. The non-fusion of the two leaves of septum pellucidum, called cavum vergae (CV), when combined with CSP, is considered the most severe form of CSP [9, 15, 16, 17].

The prevalence of CSP has been reported to be higher in individuals at risk for psychosis [10, 18]. Additionally, a meta-analysis reported a higher presence of CSP in schizophrenia patients compared to controls [19]. A large CSP is generally thought to be indirectly associated with psychotic disorders [20, 21, 22, 23, 24, 25, 26, 27, 28, 29]. A meta-analysis examining CSP in schizophrenia spectrum disorders (SSD) found that variations in small-sized CSP were not associated with SSD, but large CSP tended to be a risk factor [19]. While some recent cross-sectional studies have not found significant differences in the prevalence of large CSP between psychosis patients and healthy controls [30, 31, 32, 33, 34, 35], some studies strongly support the idea that a large CSP is associated with psychotic disorders [21, 36]. The mechanisms by which a large CSP is associated with psychiatric disorders remain uncertain. Since SP is a part of the limbic system and plays a significant role in the connections among the hypothalamus, hippocampus, amygdala, habenula, and the brainstem reticular formation [37], CSP expansion can be considered as a marker of limbic system dysgenesis [23]. Changes in CSP may reflect aetiopathogenetic brain disorders [38, 39].

However, the role of CSP in schizophrenia, its clinical course, and patients’ treatment responses remain uncertain. While antipsychotics are effective in the treatment of schizophrenia, many patients do not respond to the first antipsychotic prescribed. Patients who do not respond to a second antipsychotic treatment are typically considered treatment-resistant. Clozapine, being the only antipsychotic with proven effectiveness for treatment-resistant schizophrenia, is widely accepted as the preferred treatment option for patients who do not respond to two antipsychotic monotherapy trials. This approach is also reflected in evidence-based treatment guidelines recommending clozapine for such patients [40].

Whether CSP and/or CV serve as a risk factor for schizophrenia or are merely a reflection of neuroanatomical changes in individuals with chronic schizophrenia remains uncertain in the literature. Another significant gap in the literature is the evaluation of the relationship between clinical findings and treatment response with CSP/CV in individuals with schizophrenia. Therefore, this study assesses the relationship between CSP/CV and some clinical findings in individuals diagnosed with schizophrenia. Additionally, it tests whether CSP/CV could predict the prescribing of clozapine.

## 2. Method

Ethical approval for the study was obtained through Uskudar University Non-invasive Research Ethics Committee (Ethical approval number: 61351342/EKİM2021-37). Informed consent was obtained from each patient. A total number of one hundred and ninety patients diagnosed with schizophrenia who were admitted for inpatient treatment to NP Istanbul Brain Hospital in 2021 and underwent neuroanatomical evaluation with Magnetic Resonance Imaging (MRI) during hospitalization were included in the study. Patients with chronic neurological diseases (such as Parkinson’s disease, dementia, epilepsy, history of stroke), major pathological findings on cranial MRI (intracranial tumor, intracranial bleeding, sequelae of previous surgery, AV malformation, etc.), a history of head trauma (since previous studies have reported the development of CSP following head trauma in boxers), medical conditions that could affect cognitive functioning (such as multiple sclerosis, systemic lupus erythematosus, etc.), mental retardation, pregnancy, cranial prostheses that obstruct cranial MRI, and patients with missing data in their files were excluded. A sociodemographic data form prepared by the researchers was used in the study. Positive and Negative Syndrome Scale (PANSS) total score were recorded for each patient at admission and discharge. The presence or absence of CSP and CV was recorded as “yes” or “no”.

### 2.1 Sample 

A total of one hundred and ninety individuals aged between 18 and 65 were included in the study between January 1. 2021, and December 31. 2021.

### 2.2 Instruments 

Personal Data Form: The following demographic and clinical variables were obtained for each patient: age, gender, total years of education, employment status, marital status, income status, total duration of illness, duration of untreated illness, number of hospital admissions, history of suicide attempts, history of homicide, history of head injury, history of epilepsy, history of difficult birth, presence of additional medical conditions, smoking status, alcohol use, substance use, presence of psychiatric comorbidities, psychiatric family history, history of electroconvulsive therapy (ECT), presence of long acting injectable antipsychotic use, total number of antipsychotics used, and clozapine use.

Magnetic Resonance Imaging (MRI): Patients underwent cranial MRI scans during hospitalization as part of the study. All participants were subjected to a 1.5 Tesla Philips MRI. The images were acquired in three-dimensional planes with 5 mm thick slices without gaps, using the following parameters: Repetition Time (TR) = 11,000 ms, Echo Time (TE) = 140 ms, Inversion Time (TI) = 2800 ms, matrix size 256 × 159, Field of View (FOV) Anteroposterior (AP) × Right-left (RL) × Feet-head (FH) (mm) 230 × 188 × 142 mm, and scan duration of 2 minutes and 56 seconds. The presence of CSP (in at least one 1 mm coronal slice) was assessed by two separate psychiatrists.

Positive and Negative Syndrome Scale (PANSS): PANSS is a semi-structured interview scale with 30 items. The severity is rated on seven points [41]. It includes 30 psychiatric variables which consist of 7 positive symptom subscales, 7 negative symptom subscales, and 16 remaining general psychopathology subscales. Turkish reliability and validity study of the scale is available [42]. When the internal consistency information was examined, the Cronbach alpha coefficients of all three subscales were found to be quite high, like the coefficients found in the original study [42]. In the original study, these coefficients were 0.73, respectively; 0.83; It was found to be 0.79 [41].

### 2.3 Statistical Analysis

The data obtained in the study were analyzed using the SPSS v16.0 (IBM-SPSS Statistics, Chicago, IL, USA). Non-normally distributed data were expressed as median and interquartile range (IQR). Pearson chi-square test was used to compare categorical data. Minimum expected values were checked, and in cases where the Pearson chi-square test was not appropriate, the Fisher’s exact test was used. Normal distribution of continuous data was investigated by visual (histogram and probability graphs) and analytical methods (Shapiro-Wilk test). For the analysis of non-normally distributed continuous data the Mann-Whitney U test was used to compare continuous variables among two groups. A *p* value of <0.05 was considered statistically significant. A direct logistic regression analysis was performed on *clozapine prescription* as outcome and attitudinal predictors: Presence of CSP, PANSS total score during hospitalization, history of suicide attempts, history of homicide. First, preliminary tests were applied to identify the variables to be included in the model, and the variables to be included in the model were determined. The Hosmer-Lemeshow test was used for model fit, and it was found that the model is suitable. Logistic regression analysis was performed with enter method.

## 3. Results

The study included 190 participants, comprising of 53 females (28%) and 137 males (72%) between the ages of 18 and 65. The average age of the participants was found to be 32 (±12.6). Out of the total 190 schizophrenia patients included in the study 30.53% (n = 58) were found to have CSP and 20.53% (n = 39) CV.

Of the sample 71.6% were single (n = 136), 28.4% were married (n = 35). Among the participants 63.2% (n = 158) were employed. Considering the income status 25.3% of the participants (n = 48) described their income level as good, 46.3% as moderate, and 28.4% as poor. 25.3% of the participants (n = 48) reported a history of suicide attempts. 45.3% of the participants (n = 86) reported a history of homicide attempts. 54.7% of the participants (n = 104) reported smoking. 13.2% of the participants (n = 25) reported alcohol use. 30.6% of the participants (n = 58) reported using psychoactive substances (such as marijuana, ecstasy, cocaine, etc.) at some point in their lives. There was a history of head trauma in 10% of the participants (n = 19). Additionally 20.6% of the participants (n = 39) had a history of other medical illnesses. 65.8% of the participants (n = 125) had an additional psychiatric diagnosis. Regarding family history 50% (n = 95) reported a history of psychiatric illness in their family. In 26.3% of the participants (n = 50) there was a history of long acting injectable AP use. 14.7% of the participants (n = 28) were using clozapine. Personal data related to the participants are presented in Table 1.

**Table 1.** **Differences Between Sociodemographic Data and The Presence of CSP**.

		Presence of CSP		
		Yes	No	*p*
Gender	Male	43 (74.1%)	94 (71.2%)	0.679
	Female	15 (25.9%)	38 (28.8%)	
History of Suicide Attempts	Yes	15 (25.9%)	33 (25.0%)	0.900
	No	43 (74.1%)	99 (75.0%)	
Clozapine Prescribing	Yes	19 (32.8%)	9 (6.8%)	<0.001
	No	39 (67.2%)	123 (93.2%)	
Employment	Yes	11 (19.0%)	33 (25.0%)	0.364
	No	47 (81.0%)	99 (75.0%)	
Marital Status	Single	54 (93.1%)	101 (76.5%)	0.007
	Married	4 (6.9%)	31 (23.5%)	
Income Status	Good	19 (32.8%)	29 (22.0%)	0.085
	Poor	19 (32.8%)	35 (26.5%)	
	Moderate	20 (34.4%)	68 (51.5%)	
Homicide History	Yes	30 (51.7%)	56 (42.4%)	0.236
	No	28 (48.3%)	76 (57.6%)	
History of Head trauma	Yes	7 (12.1%)	12 (9.1%)	0.529
	No	51 (87.9%)	120 (90.9%)	
History of Other Medical Illnesses	Yes	15 (25.9%)	24 (18.2%)	0.227
	No	43 (74.1%)	108 (81.8%)	
Smoking	Yes	35 (60.3%)	69 (52.3%)	0.303
	No	23 (39.7%)	63 (47.7%)	
Alcohol Use	Yes	9 (15.5%)	16 (12.1%)	0.524
	No	49 (84.5%)	116 (87.9%)	
Substance Use	Yes	22 (37.9%)	36 (27.3%)	0.142
	No	36 (62.1%)	96 (72.7%)	
Additional Psychiatric Diagnosis	Yes	41 (70.7%)	84 (63.6%)	0.345
	No	17 (29.3%)	48 (36.4%)	
Psychiatric Family History	Yes	30 (51.7%)	65 (49.2%)	0.753
	No	28 (48.3%)	67 (50.8%)	
Long-acting Injectable AP Use	Yes	19 (32.8%)	31 (23.5%)	0.181
	No	39 (67.2%)	101 (76.5%)	
Total		58 (30.5%)	132 (69.5%)	

CSP, Cavum Septum Pellucidum; AP, Antipsychotics.

The presence of CSP was found to have no significant relationship with age (*p* = 0.679), history of suicide attempts (*p* = 0.900), employment status (*p* = 0.525), marital status (*p* = 0.120), income status (*p* = 0.085), history of homicide (*p* = 0.236), history of head trauma (*p* = 0.529), presence of additional medical illnesses (*p* = 0.227), smoking (*p* = 0.303), alcohol use (*p* = 0.524), substance use (*p* = 0.142), presence of an additional psychiatric diagnosis (*p* = 0.326), psychiatric family history (*p* = 0.753), and long-acting injectable AP use (*p* = 0.181) (Table 1). However, a significant relationship was found between the presence of CSP and clozapine prescription (*p* < 0.001) (Table 1).

The presence of CSP did not show a significant relationship with age (*p* = 0.527), total duration of education (*p* = 0.171), age of onset of the illness (*p* = 0.534), total duration of the illness (*p* = 0.915), and the duration of untreated illness (*p* = 0.528) (Table 2). The presence of CSP was found to be significantly associated with the number of hospitalizations (*p* = 0.051), the number of ECT sessions received (*p* = 0.053), and the number of AP medications used (*p* = 0.007) (Table 2). However, there was a significant association between the presence of CSP and PANSS total score at admission (*p* = 0.001) as well as PANSS total score at discharge (*p* = 0.014) (Table 2).

**Table 2.** **Differences Between Some Clinical Findings and The Presence of CSP**.

	Presence of CSP		
	Yes	No	*p*
	median ± (IQR)	median ± (IQR)	
Age	27.5 (13.8)	29.0 (13.5)	0.527
Total Duration of Education	10.4 (3.0)	11.0 (6.0)	0.171
Age of Onset	20.0 (6.0)	19.0 (9.25)	0.534
Total Duration of The Illness	8.0 (10.5)	8.0 (10.0)	0.915
The Duration of Untreated Illness	3.0 (3.75)	2.0 (4.0)	0.528
Number of Hospitalizations	2.0 (3.0)	2.0 (2.0)	0.051
Number of ECT Sessions Received	9.0 (5.0)	8.0 (10.0)	0.053
PANSS Total Score At Admission	103.5 (29.8)	89.0 (25.3)	0.001
PANSS Total Score At Discharge	51.0 (2.0)	45.0 (21.3)	0.014
Number of AP Medications Used	2.0 (1.0)	2.0 (1.0)	0.007

CSP, Cavum Septum Pellucidum; IQR, Inter Quartile Range; AP, Antipsychotic; ECT, Eectroconvulsive Therapy; PANSS, Positive and Negative Syndrome Scale.

The presence of CV was found to have no significant relationship with gender (*p* = 0.961), history of suicide attempts (*p* = 0.635), employment status (*p* = 0.727), marital status (*p* = 0.023), income status (*p* = 0.091), history of homicide attempts (*p* = 0.627), history of head trauma (*p* = 0.510), presence of additional medical illnesses (*p* = 0.183), smoking (*p* = 0.900), alcohol use (*p* = 0.944), substance use (*p* = 0.724), psychiatric family history (*p* = 0.590), and long-acting injectable AP use (*p* = 0.053) (Table 3). However, a significant relationship was observed between the presence of CV and clozapine prescribing (*p* < 0.001) (Table 3).

**Table 3.** **Differences Between Sociodemographic Data and The Presence of CV**.

		Presence of CV		
		Yes	No	*p*
Gender	Male	28 (71.8%)	109 (72.2%)	0.961
	Female	11 (28.2%)	42 (27.8%)	
History of Suicide Attempts	Yes	11 (28.2%)	37 (75.5%)	0.635
	No	28 (71.8%)	114 (24.5%)	
Clozapine Prescribing	Yes	15 (38.5%)	13 (8.6%)	<0.001
	No	24 (61.5%)	138 (91.4%)	
Homicide History	Yes	19 (48.7%)	67 (44.4%)	0.627
	No	20 (51.3%)	84 (55.6%)	
History of Head trauma	Yes	5 (12.8%)	14 (9.3%)	0.550*
	No	34 (87.2%)	137 (90.7%)	
History of Other Medical Illnesses	Yes	11 (28.2%)	28 (18.5%)	0.183
	No	28 (71.8%)	123 (81.5%)	
Smoking	Yes	21 (53.8%)	83 (55.0%)	0.900
	No	18 (46.2%)	68 (45.0%)	
Alcohol Use	Yes	5 (12.8%)	20 (13.2%)	0.944
	No	34 (87.2%)	131 (86.8%)	
Substance Use	Yes	11 (28.2%)	47 (31.1%)	0.724
	No	28 (71.8%)	104 (68.9%)	
Psychiatric Family History	Yes	18 (46.2%)	77 (51.0%)	0.590
	No	21 (53.8%)	74 (49.0%)	
Long-acting Injectable AP Use	Yes	15 (38.5%)	35 (23.2%)	0.053
	No	24 (61.5%)	116 (76.8%)	
Total		39 (20.5%)	151 (79.5%)	

CV, Cavum Vergae; AP, Antipsychotic. *Fisher’s Exact Test.

The presence of CV did not show significant correlation with age (*p* = 0.260), total duration of education (*p* = 0.017), age of onset of the illness (*p* = 0.714), total duration of the illness (*p* = 0.324), the duration of untreated illness (*p* = 0.750), the number of hospitalizations (*p* = 0.397) and the number of AP medications used (*p* = 0.252) (Table 4). The presence of CV was found to be significant correlated with the number of ECT sessions received (*p* = 0.024) (Table 4). Also, there was significant correlation between the presence of CV and PANSS total score at admission (*p* < 0.001) as well as PANSS total score at discharge (*p* = 0.016) (Table 4).

**Table 4.** **Differences Between Some Clinical Findings and The Presence of CV**.

	Presence of CV		
	Yes	No	*p*
	median ± (IQR)	median ± (IQR)	
Age	27.0 (12.0)	29.0 (13.5)	0.260
Total Duration of Education	11.0 (3.0)	11.0 (6.0)	0.017
Age of Onset of The Illness	20.0 (6.5)	19.0 (9.0)	0.714
Total Duration of The Illness	7.0 (11.5)	8.0 (10.0)	0.324
The Duration of The Illness Without Treatment	3.0 (3.0)	2.0 (4.0)	0.750
Number of Hospitalizations	2.0 (2.5)	2.0 (2.0)	0.397
Number of ECT Sessions	10.0 (5.0)	8.0 (10.0)	0.024
PANSS Total Score During Hospitalization	104.0 (33.0)	91 (28.0)	<0.001
PANSS Total Score At Discharge	54.0 (19.5)	46.0 (21.5)	0.016
Number of AP Medications	2.0 (1.0)	2.0 (1.0)	0.252

CV, Cavum Vergae; IQR, Inter Quartile Range; AP, Antipsychotics; ECT, Eectroconvulsive Therapy; PANSS, Positive and Negative Syndrome Scale.

The results of the logistic regression analysis conducted to determine whether the presence of CSP, PANSS total score, suicide history, and homicide history predict clozapine use are presented in the table. In the logistic regression model created, the presence of CSP and PANSS total score were found to predict clozapine use (respectively *p* = 0.001, *p* = 0.016). The Nagelkerke’s R^2^ value was found to be 0.167 (Table 5).

**Table 5.** **Binary Logistic Regression Analysis**.

Predictor	Odds ratio	Lower	Upper	*p*
Intercept	51.708	2.467	1083.889	0.011
Presence of CSP	4.639	1.852	11.617	0.001
PANSS total score during hospitalization	0.969	0.944	0.994	0.016
History of Suicide Attempts	1.085	0.402	2.928	0.872
History of Homicide	1.236	0.514	2.971	0.636

Nagelkerke’s R^2^ = 0.167, Omnimus Test *p* value < 0.001.

## 4. Discussion

The debate over whether schizophrenia is a single disease entity or a group of syndromes composed of multiple diseases has been ongoing for many years. Another important topic of discussion is defining treatment resistance in schizophrenia and determining the timing of clozapine introduction for a patient. Clozapine is recommended by treatment guidelines not as a first-line treatment but rather for more resistant cases or cases where extrapyramidal symptoms occur. This study showed that patients using clozapine had significantly higher rates of CSP and CV presence. Additionally, the presence of CSP/CV was found to be associated with higher hospitalization rates, higher ECT counts, higher PANSS scores at admission and at discharge, a higher number of antipsychotics used, and a higher rate of clozapine use. These associations suggest that schizophrenia patients with these malformations may have severer forms of the disorder and higher rates of antipsychotic drug resistance.

There has been a noticeable recent increase in studies examining CSP/CV prevalence in schizophrenia patients. The presence of CSP/CV of any size has been observed in both chronic [26] and first-episode psychotic patients [27]. A meta-analysis conducted by Trzesniak *et al*. [19] found that the incidence of CSP in schizophrenia patients ranged from 10.0% to 89.5%. In this study, the prevalence of CSP was found to be 30.53% and the presence of CV%, interpreted as consistent with the literature. However, there are also studies that did not find significant differences in CSP prevalence between schizophrenia patients and healthy individuals [10, 19, 20, 21, 22, 23, 30, 33, 35]. The significant differences in the prevalence of CSP/CV of any size reported in relation to schizophrenia can largely be attributed to methodological differences between studies. Some studies have used heterogeneous patient samples (including schizotypal personality disorder, schizoaffective disorder, and other psychotic disorders) [10, 23, 26, 27, 35] which can make it difficult to compare results due to variability in diagnoses across studies. Another reason for the considerable differences found among studies is the variability in the MRI scanning sequences used. In most studies, an MRI protocol with a slice thickness ranging from 1.0 mm to 1.5 mm has been used. Inconsistencies in findings across studies can also be attributed to the use of three different methodologies for evaluating CSP/CV. These three methods include the presence of CSP in at least one MRI slice; the presence of an abnormally large CSP (≥6 mm in length) [22, 23, 43]; and a grading system (ranging from 0 to 4 degrees based on the length and width of CSP) [27, 29, 35]. Some researchers have suggested that the clinical significance of CSP may be more related to its size rather than its mere presence [13, 19, 20]. Differences in the criteria used to define CSP size (small/large) also appear to have influenced study results.

The underlying mechanism behind the relationship between CSP/CV and schizophrenia has not been fully elucidated. However, since CSP is a part of the limbic system and plays a significant role in the connections among hypothalamus, hippocampus, amygdala, habenula, and the brainstem reticular formation [37], CSP may be considered a marker of limbic system dysgenesis [23]. It is well known that the fusion of SP is associated with the rapid growth of structures such as hippocampus, corpus callosum, and other midline structures [9, 44] and this has consistently been linked to schizophrenia [44]. Therefore, the presence of CSP in individuals with schizophrenia may represent an early marker of a developmental defect involving these brain regions. Based on the finding that CSP is more common in individuals at ultra-high risk for psychosis [19], it could also be considered that the changes in the cavity indicate increased susceptibility to psychosis. However, it is important to emphasize that CSP is seen in only a subgroup of individuals with schizophrenia. Therefore, this midline structural abnormality should be viewed as an early neurodevelopmental risk factor that may be associated with the presence of schizophrenia in a subset of patients, rather than a strong causative determinant of the disorder [45, 46].

We found no significant relationship between the presence of CSP/CV and gender. Similarly, a study conducted by Keshavan *et al*. [10] in 2002 did not find any association between CSP and gender. However, there is a predominance of studies indicating that males tend to have a higher incidence of CSP in any size compared to females [20, 23, 26, 28, 29, 47, 48]. This suggests the importance of considering gender in evaluating possible prenatal neurodevelopmental abnormalities, particularly those more prevalent in males [44]. Nopoulos *et al*. [23, 47] propose that an abnormality model including large CSP and decreased left temporal lobe volume in male schizophrenia cases could reflect regional, localized tissue reductions. Furthermore, they suggest that an asymmetry creating ‘different growth vectors’ in cerebral hemispheres could disrupt the normal fusion process of the septum pellucidum (i.e., ‘lateralized temporal lobe dysgenesis’). This study found no significant relationship between CSP/CV and age. It appears that the age of the subjects does not significantly affect the prevalence of CSP in the schizophrenia sample. No significant correlation between age and CSP measurements has been observed in any of the MRI studies conducted to date [10, 19]. In contrast to our study, when comparing other clinical variables between patients with and without a history of long-term hospitalization, it was found that patients who were hospitalized for a long time were significantly older than the others [24]. This study, in line with publications in the field, found no significant relationship between CSP/CV and income status [10], employment status [24], or duration of education [24]. Additionally, there was no significant association between CSP/CV and psychiatric family history in our study. However, in contrast to our findings, considering all CSP studies in schizophrenia, including postmortem examinations, there are findings associating the presence of CSP in schizophrenic patients with a family history of psychosis [48]. Furthermore, our study did not identify a significant relationship between CSP/CV and a history of suicide attempts. Similar to our findings, a study conducted by de Souza Crippa *et al*. [20] did not find a correlation between suicide rates and CSP. However, there are also studies suggesting an association between the presence of large CSP in schizophrenia and high suicide rates [49].

We found no significant relationship between CSP/CV and age of onset of the disorder. This finding is consistent with the results of studies conducted by de Souza Crippa *et al*. [20] and Fukuzako *et al*. [24]. Also there was no significant correlation between CSP/CV and total duration of illness in this study. In a study that investigated into the relationship between clinical findings and CSP in schizophrenia, the duration of illness ranged from 8 to 37 years (mean: 20.37 ± 9.19 years) [49]. In contrast, we found no significant relationship between CSP and the duration of untreated illness, as is consistent with the literature [10, 20, 21, 28, 30, 33, 49, 50, 51]. However, it has been reported that chronic schizophrenia patients exhibit a higher rate of large CSP compared to healthy individuals, while patients with first-episode psychosis show a moderate prevalence [22]. When first-episode psychosis patients were directly compared to chronic schizophrenia patients, there is evidence of volumetric differences in brain structures around CSP, such as a decrease in hippocampal volume and a decrease in the cross-sectional area of corpus callosum [52]. This suggests that controlling for the duration of illness is important in MRI studies evaluating CSP in psychotic disorders, as the fusion of septi pellucidi is likely related to the rapid growth of the hippocampus over time [9, 44], and volumetric changes in this brain structure over time may lead to changes in morphological parameters related to CSP, such as length, area, and volume.

This study found a significant relationship between CSP/CV and the number of AP’s used. In a study conducted by Fukuzako *et al*. [24], there was no significant difference in daily AP dosage between patients with and without CSP. However, in the same study, when comparisons were made between patients with and without a history of long-term hospitalization and other clinical variables, patients with long-term hospitalization had significantly higher daily AP dosage compared to others [24]. A review of the studies in the field reveals that seven studies do not mention AP use [22, 23, 26, 27, 35, 46, 50]. This lack of information regarding treatment status is probably related to the view that CSP is entirely established in early life and, therefore, CSP incidence rates would not be influenced by AP use. However, Dickey *et al*. [43] suggest that given the existing relationship between the use of these medications in schizophrenia and morphological changes in brain regions related to CSP, such as the hippocampus and corpus callosum, these medications could potentially affect CSP measurements. Additionally, this study found no significant relationship between CSP/CV and long-acting injectable AP use. This is the first study to examine the relationship between CSP presence and long-acting injectable AP use, marking a unique contribution to the field.

This study found a statistically significant relationship between the presence of CSP/CV and clozapine use in patients with schizophrenia. The presence of CSP in schizophrenic patients increased the probability of clozapine use. Also the presence of CV in schizophrenia patients increased the risk of clozapine use. To our current knowledge, there is no existing study suggesting that the presence of CSP/CV in schizophrenic patients increases the probability of clozapine use. Our study is the first in the field to shed light on this relationship. However, this relationship should be regarded not as direct causality but rather as a reflection of a more complex association. The results of the study suggest that CSP/CV predicts the clozapine use, but it remains uncertain whether the presence of CSP/CV influences the response to clozapine or it simply indicates that patients with CSP/CV require clozapine more probably than other patients. In other words, the cause-and-effect relationship of this association should be further elucidated through additional research. If CSP/CV is considered an indicator of clozapine use, this information can be taken into account by clinicians in determining treatment options for patients. Particularly in cases where resistance develops to other antipsychotics, the presence of CSP/CV may encourage the consideration of clozapine use. Furthermore, more clinical studies should be conducted to determine whether CSP/CV is one of the determinants of treatment in schizophrenia.

We found a significant relationship between CSP/CV and total PANSS score at admission. Previous studies have associated the presence of large CSP in schizophrenia with severer negative symptoms [35]. However, there are also studies that did not find a similar relationship [13, 20, 22, 24]. In a study conducted by Fukuzako *et al*. [24], no relationship was found between CSP and clinical features such as the severity of negative and positive symptoms. Additionally, in a study by de Souza Crippa *et al*. [20], it was found that CSP did not show a significant correlation with the PANSS Negative Subscale total score in the schizophrenia patient group.

A review of the existent literature reveals evidence linking the presence of CSP in schizophrenia patients to a poor prognosis [24, 53, 54, 55, 56]. Different indicators have been used in studies to determine poor prognosis. For instance, in a study by Fukuzako *et al*. [24], duration of hospitalization was considered an indicator of poor prognosis. In another study, it was found that there was a significantly higher incidence of CSP in schizophrenia patients with long-term hospitalizations [24]. Most patients with hospitalizations lasting over three years were found to have treatment-resistant psychotic symptoms. We found a significant relationship between the presence of CSP and the number of hospitalizations. Additionally, our study found a significant relationship between the presence of CSP and the total PANSS score at discharge. This finding is supported by the results of an epidemiological study by Hori *et al*. [57], which indicated that one of the factors preventing the discharge of hospitalized schizophrenia patients was the presence of treatment-resistant psychotic symptoms. The higher daily neuroleptic doses observed in patients with long-term hospitalizations may reflect these clinical aspects. The prevalence of CSP observed in patients defined as having “long-term hospitalization” was 1.8 times higher. In contrast, the prevalence of CSP in patients discharged within three years after hospitalization was equal to the prevalence in normal individuals [24]. To the best of our knowledge, our study is the first to evaluate the relationship between the number of hospitalizations, PANSS scores at both admission and discharge, and the presence of CSP, making it important for indicating that CSP may be a pathophysiological feature characterizing schizophrenia patients with a poor prognosis. We have identified a significant relationship between the presence of CSP and the number of ECT session received. ECT is an effective method used in the treatment of challenging psychiatric conditions in schizophrenia, such as suicide, aggression, acute psychotic exacerbations and catatonia. The frequency of ECT sessions and the expected response to ECT are important considerations for both patients and clinicians. Therefore, the relationship between the presence of CSP and the number of ECT sessions may be a significant factor to take into account in clinical practice. These findings could guide clinicians personalizing patients’ treatment plans and enhancing the effectiveness of ECT. However, the impact of CSP on ECT requires further research to establish a definitive cause-and-effect relationship.

## 5. Conclusions

Our study holds the distinction of being the first in the field to investigate the relationship between clozapine use and the presence of CSP/CV in schizophrenia patients. Provided that the aetiology of schizophrenia remains partially understood, and debates about its pathogenesis continue, our findings may suggest the necessity of defining a distinct subgroup within the schizophrenia spectrum characterized by the presence of relevant malformation findings. This subgroup should be characterized as having a higher level of treatment resistance, severer symptomatology, and worse prognostic features. The use of any antipsychotic drug can over time potentially modify probably not the presence or absence but the size of a CSP by alteration of possible tissue loss in surrounding structures. But the elucidation of this point would require a completely different longitudinal study design comparing the same group of patients in terms of drug use and CSP size over a rather large time span. Regarding clozapine use, another study would be required comparing 2 clozapine using samples with or without (large) CSP with each other in terms of dosage.

However, this study still has significant limitations. In this context, there is a need for longitudinal-cohort studies that can better express cause and effect to identify the conditions associated with the place of CSP and CV in the aetiogenesis of schizophrenia.

## Availability of Data and Materials

The data that support the findings of this study are available on request from the corresponding author.
